# Self-Reported Receipt of Advice and Action Taken To Reduce Dietary Sodium Among Adults With and Without Hypertension — Nine States and Puerto Rico, 2015

**DOI:** 10.15585/mmwr.mm6707a5

**Published:** 2018-02-23

**Authors:** Puthiery Va, Cecily Luncheon, Angela M. Thompson-Paul, Jing Fang, Robert Merritt, Mary E. Cogswell

**Affiliations:** ^1^Epidemic Intelligence Service, CDC; ^2^Division for Heart Disease and Stroke Prevention, National Center for Chronic Disease Prevention and Health Promotion, CDC.

Hypertension is a major cardiovascular disease risk factor (*1*,*2*). Advice given by health professionals can result in lower sodium intake and lower blood pressure (*3*).The 2017 Hypertension Guideline released by the American College of Cardiology and the American Heart Association emphasizes nonpharmacologic approaches, including sodium reduction, as important components of hypertension prevention and treatment (*4*). Data from 50,576 participants in the sodium module of the 2015 Behavioral Risk Factor Surveillance System (BRFSS) in nine states and Puerto Rico were analyzed to determine the prevalence of reported sodium reduction advice and action among participants with and without self-reported hypertension. Among participants with self-reported hypertension, adjusted prevalence of receiving sodium reduction advice from a health professional was 41.9%, compared with 12.8% among participants without hypertension. Among those with hypertension, adjusted prevalence of reported action to reduce sodium intake was 80.9% among participants who received advice and 55.7% among those who did not receive advice. Among participants without hypertension, adjusted prevalence of taking action to reduce sodium intake was 72.7% among those who received advice and 46.9% among those who did not receive advice. The provision of advice on sodium reduction by health professionals is associated with respondent action to watch or reduce sodium intake. Fewer than half of patients with hypertension received this advice from their health professionals, a circumstance that represents a substantial missed opportunity to promote hypertension prevention and treatment.

BRFSS is an annual state-based, cross-sectional telephone survey of noninstitutionalized adults aged ≥18 years. In 2015, nine states (Alabama, Indiana, Iowa, Kentucky, Maine, Nebraska, North Carolina, Oregon, and Tennessee) and Puerto Rico completed the optional sodium-related behavior module. Median survey response rate for all states and territories included in this analysis was 51.3% (range = 42.6%–59.0%) (*5*). Among 63,955 participants from jurisdictions that implemented the sodium-related behavior module, 55,857 participants completed it. After 5,281 participants with missing information on sex, age, race/ethnicity, education, smoking status, body mass index, and reported comorbidities were excluded, data from 50,576 respondents (90.5% of all participants) were analyzed. Prevalence of sodium reduction advice and action was estimated by self-reported hypertension status. Hypertension was defined as an affirmative response to the question “Have you ever been told by a doctor, nurse, or other health professional that you have high blood pressure?” Women who answered “yes” but “only during pregnancy,” as well as those who were told that they were “borderline high or pre-hypertensive” were not included. Receiving health professional advice to reduce sodium intake was defined by an affirmative response to the question “Has a doctor or other health professional ever advised you to reduce sodium or salt intake?” Action to reduce sodium intake was defined by an affirmative response to the question “Are you currently watching or reducing your sodium or salt intake?”

Descriptive analyses were used to examine population characteristics by hypertension status. Multiple variable logistic regression was used to examine characteristics associated with advice and action and to estimate prevalence and 95% confidence intervals using predicted marginals adjusted for selected covariates (*6*). Covariates included sociodemographic characteristics (geographic location, sex, age/ethnicity, race, and education) and cardiovascular disease risk factors (smoking, obesity status, and reported associated comorbidities [diabetes, kidney disease, myocardial infarction, coronary heart disease, or stroke]). All estimates used sampling weights to account for the complex survey design and nonresponse. Chi-square tests were used to compare prevalence estimates. P-values <0.05 were considered statistically significant.

Participants with self-reported hypertension differed significantly from participants without hypertension for all characteristics examined (p<0.05 for all characteristics) (Table 1). Among participants with hypertension compared with those without hypertension, more participants were male (51.0% versus 48.6%), aged ≥65 years (37.0% versus 11.9%), non-Hispanic black (13.9% versus 9.6%), had less than a high school education (19.3% versus 11.6%), were current or former smokers (51.0% versus 41.0%), had obesity (45.1% versus 25.0%), or reported ≥1 comorbidity (39.8% versus 8.9%).

**TABLE 1:** Unadjusted prevalence* of selected characteristics of adults aged ≥18 years by hypertension^†^ status — Behavioral Risk Factor Surveillance System, nine states and Puerto Rico, 2015

Characteristic	Hypertension status % (95% CI)^§^	
	Self-reported hypertension (n = 22,606)	No self-reported hypertension (n = 27,970)
**Jurisdiction**		
Alabama	11.9 (11.4–12.3)	10.0 (9.6–10.4)
Indiana	12.1 (11.5–12.8)	14.5 (13.9–15.1)
Iowa	5.5 (5.3–5.8)	7.2 (6.9–7.5)
Kentucky	10.2 (9.8–10.7)	9.1 (8.7–9.5)
Maine	3.1 (2.9–3.3)	3.4 (3.3–3.6)
Nebraska	3.5 (3.3–3.7)	4.6 (4.4–4.8)
North Carolina	21.3 (20.5–22.1)	20.9 (20.3–21.5)
Oregon	7.2 (6.6–7.8)	9.6 (9.0–10.1)
Tennessee	14.7 (14.0–15.4)	12.1 (11.6–12.7)
Puerto Rico	10.6 (10.1–11.0)	8.6 (8.2–8.9)
**Sex**		
Male	51.0 (49.9–52.0)	48.6 (47.6–49.5)
Female	49.0 (48.0–50.1)	51.5 (50.5–52.4)
**Age group (yrs)**		
18–64	63.0 (62.1–63.9)	88.1 (87.6–88.5)
≥65	37.0 (36.1–37.9)	11.9 (11.5–12.4)
**Race/Ethnicity**		
White, non-Hispanic	70.5 (69.6–71.5)	72.5 (71.7–73.3)
Black, non-Hispanic	13.9 (13.1–14.7)	9.6 (9.0–10.3)
Other, non-Hispanic	2.9 (2.5–3.4)	4.1 (3.7–4.5)
Hispanic	12.7 (12.1–13.3)	13.8 (13.2–14.4)
**Education**		
Less than high school	19.3 (18.3–20.2)	11.6 (10.9–12.4)
High school	32.2 (31.2–33.1)	29.1 (28.2–30.0)
Some college	29.7 (28.8–30.7)	33.1 (32.2–34.0)
College or more	18.9 (18.2–19.6)	26.2 (25.5–26.9)
**Smoking status**		
Current and former smoker	51.0 (50.0–52.0)	41.0 (40.1–41.9)
Never smoker	49.0 (48.0–50.0)	59.0 (58.1–59.9)
**Obesity status^¶^**		
No	55.0 (53.9–56.0)	75.0 (74.1–75.8)
Yes	45.1 (44.0–46.1)	25.0 (24.2–25.9)
Comorbidities**		
No	60.2 (59.1–61.2)	91.1 (90.6−91.5)
Yes	39.8 (38.8–40.9)	8.9 (8.5–9.4)

**Abbreviation**: CI = confidence interval.

* Unadjusted prevalence estimates weighted for survey design and nonresponse.

^†^ Hypertension was defined as an affirmative response to the question “Have you ever been told by a doctor, nurse, or other health professional that you have high blood pressure?”

^§^ p-value <0.05 for differences (chi-square test) in percent distribution of covariates between participants with reported hypertension and without reported hypertension, accounting for complex survey design and weighted.

^¶^ Obesity defined as body mass index ≥30 kg/m^2^.

** Includes self-reported diabetes, kidney disease, myocardial infarction, coronary heart disease, or stroke.

After adjusting for sociodemographic and cardiovascular risk factors, the prevalence of having received sodium reduction advice was 41.9% among participants with hypertension and 12.8% among those without hypertension (Table 2) (p<0.05 for difference overall and in each subgroup). Among participants with hypertension, the adjusted prevalence of receiving advice varied significantly by geographic location, ranging from 32.3% (Oregon) to 56.7% (Puerto Rico), and by sex, race/ethnicity, obesity status, and reported presence of ≥1 comorbidity, but not by age, level of education, or smoking status. By covariate, receipt of advice was higher, for example, among participants who were female (43.0%) versus male (40.8%); non-Hispanic black (54.1%) and Hispanic (46.1%) versus non-Hispanic white (39.1%); who had obesity (46.6%) versus those who did not have obesity (40.2%); and who had ≥1 comorbidity (53.4%) versus no comorbidity (40.0%) (Table 2). Among participants without hypertension, the prevalence of receiving advice ranged from 9.4% (Oregon) to 22.0% (Puerto Rico). Prevalence of receiving advice varied significantly by selected covariate (p<0.05), except sex. By covariate, the adjusted prevalence of advice was higher among non-Hispanic black (16.9%) and Hispanic participants (16.8%) than among non-Hispanic white participants (10.8%), among participants with a high school diploma (14.0%) or less than a high school education (14.9%) than among those with college or more (10.5%), among current or former smokers (13.9%) than among never smokers (11.9%), among those who had obesity (17.4%) versus those who did not (10.6%), and among those who reported ≥1 comorbidity (26.6%) than among those who did not (10.0%) (Table 2).

**TABLE 2:** Adjusted* percentage of adults aged ≥18 years who reported receiving advice to reduce their sodium intake, by hypertension^†^ status — Behavioral Risk Factor Surveillance System, nine states and Puerto Rico, 2015

Characteristic	Reported receiving advice					
	Self-reported hypertension^§^			No self-reported hypertension		
	No.	% (95% CI)	p-value^¶^	No.	% (95% CI)	p-value^¶^
**Total**	**22,606**	**41.9 (40.8–43.0)**	**—**	**27,970**	**12.8 (12.1–13.4)**	**—**
**Jurisdiction**						
Alabama	3,048	39.8 (37.3–42.4)	<0.05	3,159	12.7 (11.2–14.4)	<0.05
Indiana	2,043	43.1 (39.9–46.3)		2,613	11.5 (9.8–13.5)	
Iowa	1,884	37.9 (35.1–40.9)		2,857	11.3 (9.7–13.1)	
Kentucky	3,372	40.3 (37.5–43.2)		3,473	11.2 (9.6–13.0)	
Maine	1,941	44.8 (41.8–47.8)		2,740	13.6 (11.8–15.7)	
Nebraska	2,758	33.3 (30.7–36.0)		4,376	9.6 (8.1–11.3)	
North Carolina	2,152	43.7 (41.1–46.4)		2,909	12.1 (10.7–13.7)	
Oregon	744	32.3 (28.2–36.7)		1,188	9.4 (7.0–12.6)	
Tennessee	2,210	40.3 (37.1–43.6)		2,154	11.7 (9.8–13.9)	
Puerto Rico	2,454	56.7 (51.2–62.1)		2,501	22.0 (18.5–26.0)	
**Sex**						
Male	9,548	40.8 (39.3–42.4)	<0.05	11,582	12.9 (11.9–13.8)	0.980
Female	13,058	43.0 (41.5–44.4)		16,388	12.7 (11.9–13.5)	
**Age group (yrs)**						
18–64	11,264	42.7 (41.3–44.1)	0.582	21,439	11.4 (10.7–12.1)	<0.05
≥65	11,342	42.6 (41.1–44.1)		6,531	20.2 (18.7–21.7)	
**Race/Ethnicity**						
White, non-Hispanic	16,928	39.1 (37.7–40.6)	<0.05	22,016	10.8 (10.1–11.6)	<0.05
Black, non-Hispanic	2,398	54.1 (50.6–57.6)		1,769	16.9 (14.4–19.6)	
Other, non-Hispanic	570	40.2 (33.9–46.9)		881	15.3 (10.8–21.3)	
Hispanic	2,710	46.1 (41.0–51.3)		3,304	16.8 (14.2–19.7)	
**Education**						
Less than high school	2,670	43.0 (40.0–46.0)	0.377	1,848	14.9 (13.0–17.0)	<0.05
High school	7,610	41.8 (40.1–43.6)		7,882	14.0 (12.9–15.3)	
Some college	6,128	41.3 (39.4–43.2)		7,966	12.2 (11.1–13.4)	
College or more	6,198	42.9 (41.0–44.8)		10,274	10.5 (9.6–11.4)	
**Smoking status**						
Current and former smoker	10,938	41.2 (39.7–42.8)	0.245	11,358	13.9 (13.0–15.0)	<0.05
Never smoker	11,668	42.7 (41.2–44.2)		16,612	11.9 (11.1–12.8)	
Obesity Status**						
No	12,966	40.2 (38.8–41.6)	<0.05	21,037	10.6 (10.0–11.3)	<0.05
Yes	9,640	46.6 (44.9–48.2)		6,933	17.4 (16.1–18.8)	
**Comorbidities^††^**						
No	13,231	40.0 (38.7–41.4)	<0.05	24,674	10.0 (9.4–10.6)	<0.05
Yes	9,375	53.4 (51.7–55.1)		3,296	26.6 (24.4–29.0)	

**Abbreviation:** CI = confidence interval.

* Adjusted prevalence estimates were estimated from marginal predictions of separate multiple logistic regression models for each covariate with a term for the interaction between the covariate (e.g., sex) and hypertension status adjusted for all the other covariates in the table, accounting for survey design and survey weights. Significant interactions occurred between hypertension status and age, race/ethnicity, education, smoking status, obesity status, and comorbidities.

^†^ Hypertension was defined as an affirmative response to the question “Have you ever been told by a doctor, nurse, or other health professional that you have high blood pressure?”

^§^ Across all participating locations and selected covariates, a higher prevalence of advice was reported among participants with hypertension compared with those without hypertension (p–value <0.05).

^¶^ p-value obtained by Wald F test and p-value <0.05 were used to identify statistically significant differences in prevalence of advice among subgroups with hypertension and without hypertension.

** Obesity defined as body mass index ≥30 kg/m^2^.

^††^ Includes self-reported diabetes, kidney disease, myocardial infarction, coronary heart disease, or stroke.

Overall, participants with hypertension who received advice had the highest adjusted prevalence of taking action to reduce sodium intake (80.9%), followed by those without hypertension who received advice (72.7%), those with hypertension who did not receive advice (55.7%), and those without hypertension who did not receive advice (46.9%) (p<0.05 for overall comparison across the four groups) (Table 3).

**TABLE 3:** Adjusted* percentage of adults aged ≥18 years who report taking action to reduce their sodium intake, by receipt of advice to reduce sodium intake and self-reported hypertension^†^ status — Behavioral Risk Factor Surveillance System, nine states and Puerto Rico, 2015

Characteristic	Took action to reduce sodium intake											
	Self-reported hypertension						No self-reported hypertension					
	Advice			No advice			Advice			No advice		
	No.	% (95% CI)	p-value^§^	No.	% (95% CI)	p-value^§^	No.	% (95% CI)	p-value^§^	No.	% (95% CI)	p-value^§^
**Total**	**10,900**	**80.9 (79.5–82.2)**	**—**	**11,706**	**55.7 (54.2–57.2)**	**—**	**3,346**	**72.7 (70.1–75.2)**	**—**	**24,624**	**46.9 (45.9–47.9)**	**—**
**Jurisdiction**												
Alabama	1,481	80.5 (77.2–83.5)	<0.05	1,567	56.5 (53.0–59.9)	<0.05	424	75.3 (68.7–80.9)	0.330	2,735	45.2 (42.7–47.8)	<0.05
Indiana	956	82.8 (79.1–86.0)		1,087	51.4 (46.9–55.8)		302	71.3 (62.3–78.9)		2,311	47.9 (45.0–50.9)	
Iowa	763	82.4 (78.3–85.8)		1,121	52.3 (48.5–56.0)		278	69.7 (61.1–77.2)		2,579	42.1 (39.6–44.7)	
Kentucky	1,664	76.1 (71.9–79.9)		1,708	54.3 (50.4–58.3)		402	72.2 (65.2–78.3)		3,071	42.2 (39.4–45.1)	
Maine	908	85.0 (81.6–87.8)		1,033	57.9 (54.0–61.8)		306	74.9 (67.9–80.8)		2,434	46.0 (43.2–48.7)	
Nebraska	1,063	82.9 (79.3–85.9)		1,695	51.0 (47.1–54.8)		344	68.3 (58.4–76.7)		4,032	39.2 (36.9–41.6)	
North Carolina	1,095	83.9 (80.6–86.8)		1,057	59.2 (55.3–62.9)		321	71.4 (64.6–77.3)		2,588	49.5 (47.2–51.8)	
Oregon	268	83.8 (77.3–88.7)		476	49.7 (43.4–55.9)		82	71.6 (55.0–83.9)		1,106	37.2 (33.4–41.1)	
Tennessee	1,024	78.9 (74.4–82.7)		1,186	56.8 (52.5–61.1)		238	81.1 (72.4–87.6)		1,916	51.4 (48.0–54.7)	
Puerto Rico	1,678	81.3 (77.4–84.8)		776	62.2 (56.0–68.1)		649	74.7 (68.9–79.8)		1,852	56.7 (51.8–61.4)	
**Sex**												
Male	4,467	79.3 (77.3–81.2)	<0.05	5,081	51.0 (48.7–53.2)	<0.05	1,419	70.9 (66.9–74.7)	0.077	10,163	43.1 (41.6–44.6)	<0.05
Female	6,433	82.4 (80.6–84.0)		6,625	60.5 (58.6–62.4)		1,927	74.5 (71.2–77.7)		14,461	50.6 (49.2–51.9)	
**Age group (yrs)**												
18–64	5,519	79.5 (77.7–81.2)	<0.05	5,745	55.1 (53.1–57.1)	<0.05	2,230	69.8 (66.6–72.8)	<0.05	19,209	44.5 (43.3–45.6)	<0.05
≥65	5,381	85.9 (84.3–87.3)		5,961	61.2 (59.2–63.1)		1,116	84.1 (80.5–87.1)		5,415	56.6 (54.6–58.6)	
**Race/Ethnicity**												
White, non-Hispanic	7,381	80.3 (78.7–81.9)	<0.05	9,547	53.1 (51.3–54.9)	<0.05	2,177	73.3 (70.1–76.3)	0.281	19,839	43.5 (42.2–44.7)	<0.05
Black, non-Hispanic	1,449	87.7 (84.5–90.3)		949	71.6 (66.7–76.0)		312	77.4 (69.0–84.1)		1,457	61.3 (57.6–65.0)	
Other, non-Hispanic	270	81.2 (67.5–90.0)		300	49.5 (39.6–59.5)		97	84.0 (70.9–91.9)		784	46.3 (41.1–51.7)	
Hispanic	1,800	79.8 (75.8–83.3)		910	57.8 (51.2–64.2)		760	70.2 (64.1–75.7)		2,544	53.4 (49.1–57.7)	
**Education**												
Less than high school	1,527	77.0 (72.9–80.5)	0.079	1,143	55.3 (50.7–59.7)	0.347	380	66.5 (58.5–73.6)	0.269	1,468	46.6 (42.7–50.5)	0.641
High school	3,684	80.4 (78.1–82.5)		3,926	54.9 (52.4–57.5)		1,088	74.0 (69.3–78.1)		6,794	46.3 (44.5–48.2)	
Some college	2,885	83.7 (81.4–85.7)		3,243	57.6 (55.0–60.2)		916	72.0 (67.0–76.5)		7,050	47.5 (45.7–49.2)	
College or more	2,804	81.0 (78.5–83.2)		3,394	53.9 (51.3–56.4)		962	76.3 (72.1–80.0)		9,312	47.1 (45.6–48.7)	
**Smoking status**												
Current and former smoker	5,146	79.9 (77.9–81.8)	<0.05	5,792	55.0 (52.8–57.1)	0.514	1,454	72.0 (68.2–75.5)	0.210	9,904	46.0 (44.5–47.6)	0.172
Never smoker	5,754	81.8 (80.0–83.4)		5,914	56.3 (54.1–58.3)		1,892	73.4 (69.7–76.8)		14,720	47.6 (46.2–48.9)	
**Obesity status^¶^**												
No	5,843	82.5 (80.9–84.1)	<0.05	7,123	53.6 (51.7–55.6)	<0.05	2,160	73.0 (69.6–76.2)	0.971	18,877	45.8 (44.6–46.9)	<0.05
Yes	5,057	79.8 (77.7–81.7)		4,583	59.3 (56.9–61.6)		1,186	72.6 (68.4–76.3)		5,747	49.4 (47.4–51.4)	
Comorbidities**												
No	5,520	80.1 (78.3–81.8)	<0.05	7,711	55.2 (53.4–57.0)	<0.05	2,423	70.3 (67.2–73.2)	<0.05	22,251	45.1 (44.0–46.2)	<0.05
Yes	5,380	84.6 (82.8–86.2)		3,995	59.8 (57.2–62.3)		923	82.1 (77.8–85.8)		2,373	55.0 (51.9–58.1)	

**Abbreviation:** CI = confidence interval.

* Adjusted prevalence estimates were estimated from marginal predictions of separate multiple logistic regression models for each covariate with a term for the interaction between the covariate (e.g., sex) and hypertension status adjusted for all the other covariates in the table. Significant interactions occurred between the hypertension and advice with state, age, race/ethnicity, obesity status, and comorbidities.

^†^ Hypertension was defined as an affirmative response to the question “Have you ever been told by a doctor, nurse, or other health professional that you have high blood pressure?”

^§^ p-value obtained by Wald F test and p<0.05 were used to identify statistically significant differences in prevalence of action among subgroups with hypertension and without hypertension by receipt of advice.

^¶^ Obesity defined as body mass index ≥30 kg/m^2^

** Includes self-reported diabetes, kidney disease, myocardial infarction, coronary heart disease, or stroke.

## Discussion

In 2015, fewer than half (42%) of BRFSS participants with self-reported hypertension from nine states and Puerto Rico (range = 32% [Oregon] to 57% [Puerto Rico]) reported receiving sodium reduction advice from a health professional independent of sociodemographic characteristics and cardiovascular disease risk factors. Among respondents without hypertension, 13% reported receiving advice to reduce sodium intake (range = 9% [Oregon] to 22% [Puerto Rico]). Yet, among participants with hypertension who received advice, 81% reported taking action to reduce sodium, compared with 56% of those with hypertension who did not receive advice. Similarly, among participants without hypertension 73% of those who received advice to reduce sodium intake reported taking action to reduce sodium, compared with 47% of those who did not receive advice. In this analysis, among participants with and without hypertension, receiving sodium reduction advice from a health professional was associated with reported respondent action to watch or reduce sodium intake.

This study provides the most recent multistate BRFSS data on sodium reduction advice and action. Comparing these results with previously published BRFSS and other data are difficult, given differences in sample size, number of states, and analytic method. Despite these differences, results were generally consistent with previous studies that found respondents with hypertension were more likely to receive advice and take action (*7*) and that the prevalence of taking action was highest among those who received advice (*8*).

Fewer than half of adults with hypertension in most locations, and even fewer adults without hypertension, reported receiving sodium reduction advice. Geographic patterns of prevalence of receiving advice appears to correspond with the pattern of self-reported “high blood pressure” diagnosis. For example, Puerto Rico, which had a prevalence of self-reported hypertension (42.2%) substantially higher than the national prevalence of 30.9% (*9*), had one of the highest prevalences of receiving advice and taking action. Similar to previous reports, in this study, the prevalence of receiving advice was significantly higher among persons with hypertension and obesity or other cardiovascular disease–associated comorbidity than among those with hypertension without these other risk factors. However, among adults with an elevated risk for cardiovascular disease, but without hypertension, reported advice to reduce sodium intake was <30%. Also consistent with earlier findings, more adults who received advice from a health professional to reduce sodium intake reported watching or reducing their sodium intake, irrespective of hypertension status or cardiovascular risk factors (*7*). Self-reported action to watch or reduce sodium intake might not result in achieving clinically meaningful sodium reduction (*10*); however, these findings suggest that a health professional’s advice can significantly affect awareness.

The findings in this report are subject to at least three limitations. First, BRFSS data are self-reported and subject to recall and social desirability bias, which affects prevalence estimates. Second, questions from BRFSS do not provide the extent of health professional advice or verify or detail the types of actions taken by respondents who report actively watching or reducing their sodium intake. Therefore, these questions might serve as a proxy for awareness of the need for sodium reduction rather than a measure of behavior change. Finally, responses were limited to nine states and Puerto Rico that elected to apply the sodium module during the 2015 BRFSS, and where response rates were approximately 50%; therefore, these results might not be generalizable to all U.S. adults and could be subject to response bias. Despite limitations, this report estimates sodium reduction advice and action using the latest BRFSS data and might provide a baseline for current practice as well as demonstrate opportunities for increasing the advice provided.

The findings from this analysis indicate that a higher percentage of BRFSS participants who reported receiving sodium reduction advice from a health professional reported taking action, across hypertension status and cardiovascular risk groups, underscoring the importance of health professional advice on potentially influencing sodium reduction awareness and behavior. Yet, fewer than half of respondents with self-reported hypertension and fewer respondents without hypertension reported receiving advice. In accordance with the 2017 hypertension guidelines (*4*) encouraging lifestyle modification, health professionals can encourage healthy food choices and support consumer and population efforts to reduce sodium intake, highlighting a potential opportunity for hypertension prevention and treatment.

**Summary**

**What is already known about this topic?**

Hypertension is a major cardiovascular disease risk factor for which sodium reduction can be beneficial. Provision of sodium reduction advice by health professionals to persons with hypertension reduces their reported sodium intake.

**What is added by this report?**

Among participants with self-reported hypertension, the prevalence of receiving advice to reduce sodium intake from a health professional was 42% compared with 13% among participants without hypertension. Among those with hypertension, 81% of those who received advice to reduce sodium intake reported taking action to reduce sodium intake, compared with 56% of those with hypertension who did not receive this advice.

**What are the implications for public health practice?**

Most patients do not receive clinical advice to reduce sodium intake. Increasing the percentage of patients who receive this advice from their health care provider might provide increased opportunities for hypertension prevention and treatment.
