# *Erratum:* Vol. 67, No. 7

**DOI:** 10.15585/mmwr.mm6713a5

**Published:** 2018-04-06

In the report “Self-Reported Receipt of Advice and Action Taken To Reduce Dietary Sodium Among Adults With and Without Hypertension — Nine States and Puerto Rico, 2015,” on page 225, the third sentence of the second paragraph should have read “Median survey response rate for all states and territories included in this analysis was 51.3% (**range = 38.6%–59.0%**) (*5*).”

